# Exploring genetic diversity and population structure of a large grapevine (*Vitis vinifera* L.) germplasm collection in Türkiye

**DOI:** 10.3389/fpls.2023.1121811

**Published:** 2023-05-10

**Authors:** Hilal Betul Kaya, Yıldız Dilli, Tulay Oncu-Oner, Akay Ünal

**Affiliations:** ^1^ Department of Bioengineering, Manisa Celal Bayar University, Manisa, Türkiye; ^2^ Republic of Türkiye Ministry of Agriculture and Forestry, Viticulture Research Institute, Manisa, Türkiye

**Keywords:** *Vitis Vinifera* L., genetic characterization, linkage disequilibrium, divergent loci, genotyping by sequencing

## Abstract

Grapevine (*Vitis Vinifera* L.) has been one of the significant perennial crops in widespread temperate climate regions since its domestication around 6000 years ago. Grapevine and its products, particularly wine, table grapes, and raisins, have significant economic importance not only in grapevine-growing countries but also worldwide. Grapevine cultivation in Türkiye dates back to ancient times, and Anatolia is considered one of the main grapevine migration routes around the Mediterranean basin. Turkish germplasm collection, conserved at the Turkish Viticulture Research Institutes, includes cultivars and wild relatives mainly collected in Türkiye, breeding lines, rootstock varieties, and mutants, but also cultivars of international origin. Genotyping with high-throughput markers enables the investigation of genetic diversity, population structure, and linkage disequilibrium, which are crucial for applying genomic-assisted breeding. Here, we present the results of a high-throughput genotyping-by-sequencing (GBS) study of 341 genotypes from grapevine germplasm collection at Manisa Viticulture Research Institute. A total of 272,962 high-quality single nucleotide polymorphisms (SNP) markers on the nineteen chromosomes were identified using genotyping-by-sequencing (GBS) technology. The high‐density coverage of SNPs resulted in an average of 14,366 markers per chromosome, an average polymorphism information content (PIC) value of 0.23 and an expected heterozygosity (He) value of 0.28 indicating the genetic diversity within 341 genotypes. LD decayed very fast when *r^2^* was between 0.45 and 0.2 and became flat when *r^2^* was 0.05. The average LD decay for the entire genome was 30 kb when *r^2 = ^* 0.2. The PCA and structure analysis did not distinguish the grapevine genotypes based on different origins, highlighting the occurrence of gene flow and a high amount of admixture. Analysis of molecular variance (AMOVA) results indicated a high level of genetic differentiation within populations, while variation among populations was extremely low. This study provides comprehensive information on the genetic diversity and population structure of Turkish grapevine genotypes.

## Introduction

Grapevine (*Vitis vinifera* L.) is one of the most important fruit crops worldwide (Jaillon et al., 2007). Besides its broad uses in wine industries, this fruit crop has been used to produce raisins, fresh fruits, juices, and leaves. In addition, grapes are an important source of secondary metabolites used in the cosmetic, food, and pharmaceutical industries (Ali et al., 2010). The beneficial effects of these secondary metabolites on several diseases have been reported in some studies (Folts, 2002; Tyagi et al., 2003; Tenkumo et al., 2020). In 2020, the total area of harvested grapes worldwide was 7 million hectares, with 78 million tons of grapes (FAOSTAT, 2018). China is the largest producer of grapes in the world, followed by Italy, the USA, Spain, and France. Türkiye is a critical producer in the grape market, ranked 6th in the world in 2020 according to total grape production.

The archaeological record suggests that grapevine domestication began in southeastern Anatolia (the Asian part of Türkiye) or the region known as Transcaucasian about 6000 to 7000 years ago before grapevine cultivars spread throughout neighboring regions of Europe and Northern Africa (Zohary et al., 2012; Mc Govern, 2003). During the spreading period *via* different routes, high genetic diversity among grapevine cultivars is a result of sexual reproduction (Vouillamoz and Grando, 2006), (Myles et al., 2011), and spontaneous mutations (Margaryan et al., 2021). According to Vitis international variety catalog (Maul and Töpfer, 2015), there are more than 12,000 documented grapevine cultivars. Still, only a small number of cultivars have economic importance worldwide (Bouby et al., 2013), and growers have been mainly focused on them. Although the rapid spread of grapevine disease phylloxera has caused a significant decrease in grapevine genetic diversity, the elimination of old and local cultivars from vineyards due to their low-quality characteristics over time has also led to a gradual reduction in genetic resources (Tello et al., 2019a).

Grapevine germplasm collections, including cultivars, wild relatives, breeding lines, rootstock varieties, and mutants, have been constructed for characterization, conservation, use, and development of grapevine genetic resources in the leading grapevine-growing countries such as Spain, France, Germany, Italy, and the USA (Martín et al., 2006; Lacombe et al., 2007; Cipriani et al., 2010; Maul et al., 2012; Žulj Mihaljević et al., 2020). In addition, these germplasm collections have been prevalently used in molecular studies, including identification (Laucou et al., 2011; Laucou et al., 2018), molecular characterization (Lopes et al., 1999; Nicolas et al., 2016), and mapping studies (Myles et al., 2011; Laucou et al., 2018; Guo et al., 2019). In Türkiye, there are two Viticulture Research Institutes in Tekirdağ and Manisa, Tekirdağ Viticulture Research Institute hold approximately 1200 genotypes of cultivars and some important breeding lines with wild relatives (Söylemezoğlu et al., 2016). With the efforts of protection of Turkish Germplasm collection, Manisa Viticulture Research Institutes was established in 1930 and started to conduct surveys to preserve Turkish grapes in the Aegean region. In the following years, some other important genotypes, including national and international cultivars, were transferred from Ege University and Aegean Agricultural Research Institute, one of the government research institutes of TAGEM (General Directorate of Agricultural Research and Policies).

Türkiye is one of the most favorable locations for viticulture in terms of ecological conditions, and grape production ranks first in fruit production, making a significant contribution to Türkiye’s economy. However, grapevine cultivation is mainly concentrated in the western part of the country, especially in the Aegean region. Mediterranean and Marmara regions rank second and third, respectively, based on both areas of vineyards and grape production. Although vineyards are spread throughout the country, the other regions cover a relatively small area for grapevine production. In Türkiye, Manisa is the leading grape producer city, with 809 thousand hectares of vineyard areas, accounting for approximately 38% of the total output in the country (TUIK, 2019). In the exploration survey of grapevine genetic resources of Türkiye in 2021, 1439 different genotypes were protected in the Turkish National Grapevine Genetic Reserve in Tekirdağ Viticulture Research Institute.

Characterizing the genetic diversity and population structure of grapevine germplasms is crucial to provide valuable genetic resources for grapevine genetic improvement and to protect and to develop this vital crop (Emanuelli et al., 2013; De Oliveira et al., 2020). Unfortunately, mislabeling of genotypes as homonyms or synonyms is widespread among grapevine genotypes, leading to problems in classification. For a sustainable viticulture industry, extensive genetic diversity should be identified and maintained to be able to develop new grape cultivars with desired traits through marker-assisted breeding (Di Gaspero and Cattonaro, 2010; Myles et al., 2011).

Ampelographic identification, including the morphological characteristics of leaves, shoot tips, branches, fruit bunches, and berries, has been traditionally used to characterize grapevine cultivars (Topfer et al., 2009). However, the molecular characterization of plant germplasm is the preferred approach to identify genetic diversity and variation within the population (Gago et al., 2022). Information regarding genetic diversity, population structure, and gene flow are essential for accelerating the development of efficient breeding strategies, and DNA markers are powerful tools for identifying and characterizing diverse genotypes (Myles et al., 2011; De Oliveira et al., 2020). In addition, DNA markers provide useful information in theoretical and applied research fields for grapevine breeding, such as cultivar identification, determination of genetic diversity, paternity analyses, characterization of large grapevine germplasms, linkage map construction, and detection of marker-trait associations *via* QTL (Quantitative Trait Locus) and association mapping (Tomić et al., 2013). Although various types of molecular markers, including RFLP (Restriction fragment length polymorphism), RAPD (Random Amplified Polymorphic DNA), SSR (Simple Sequence Repeats), and SNP (Single Nucleotide Polymorphism), have been utilized in grapevine (Lodhi et al., 1995; Dalbó et al., 2000; Adam-Blondon et al., 2004), SSRs have been extensively used because of their high polymorphism, codominant nature, and reproducibility (Riaz et al., 2004; This et al., 2004). However, using SSR as a genotyping platform for large-scale germplasm screening has some drawbacks, including being time-consuming, labor-intensive, and low-throughput (Deschamps et al., 2012).

Next-generation sequencing (NGS) has enabled the discovery of thousands of markers in large and diverse germplasm collections (Crossa et al., 2013). NGS-based genome-wide SNP (single nucleotide polymorphism) markers have emerged as a powerful molecular tool for germplasm characterization and population structure studies. Genotyping by sequencing (GBS) is a simple and relatively inexpensive technique that reduces genome complexity using restriction enzymes for NGS-based SNP identification. With this advantage, GBS has been implemented in various woody perennial species such as olive (D’Agostino et al., 2018; Kaya et al., 2019), peach (Bielenberg et al., 2015), apple (Gardner et al., 2014), sweet cheery (Guajardo et al., 2015), almond (Goonetilleke et al., 2018), and oil palm (Teh et al., 2016), and found to be effective for high-throughput genotyping. The first report on the use of GBS in grapevine was to discover SNPs in an F1 segregation population to analyze the inheritance of powdery mildew resistance (Barba et al., 2014). However, the same group reports high rates of missingness and heterozygote under-calling. Because of the highly heterozygous and diverse grapevine genome, they developed a modular approach to overcome these problems. Recently, GBS was applied for clarification of evolutionary relationships among North American Vitis species (Klein et al., 2018), characterization of some accessions in the USDA (United States Department of Agriculture) Vitis germplasm collections (Klein et al., 2018), and identification of marker-trait associations in grapevine by GWAS (Genome-wide association studies) (Guo et al., 2019; Flutre et al., 2020) and QTL mapping (Barba et al., 2014) which demonstrated the suitability of GBS for the high-throughput genotyping in grapevine. RAD (Restriction-site Associated DNA)-sequencing, a similar approach to GBS, has also been implemented to characterize the relatedness between wild and cultivated grapevine in a germplasm collection (Marrano et al., 2017). The present study is the first report on discovering GBS-generated SNP markers in a diverse collection of Turkish grapevine genotypes. In this study, we aimed (1) to characterize 341 grapevine genotypes originating from different regions of Turkey, mostly Aegean Region genotypes, and some other countries, (2) to detect synonymies, homonymies, and misnaming and, (3) to assess the level of genetic diversity. The results not only indicate the nature and extent of the genetic diversity in the grapevine genotypes but also estimate population structure and characterize the LD pattern.

## Materials and methods

### Plant materials

A total of 341 genotypes from grapevine germplasm collection in Manisa Viticulture Research Institute (38°N, 27°E) were genetically characterized in this study. This grapevine sample set includes genotypes from Aegean Region, Marmara Region, Mediterranean Region, Central Anatolia, Eastern Anatolia Region, Black Sea Region, Southeastern Anatolia Region as well as 80 genotypes from unknown locations in Türkiye and nine genotypes from France, nine genotypes from Italy, one genotype from Spain, one genotype from Greece, one genotype from Uzbekistan, three genotypes from the USA and two unknown genotypes. A list of all samples and their specific codes is available in **Supplementary Table S1**.

### DNA extraction, library preparation, and sequencing

DNA was extracted from young leaves using the CTAB (Cetyltrimethylammonium bromide) protocol (Lodhi et al., 1994). The quality and quantity of genomic DNA were evaluated using the Qubit™ Fluorometer 3.0 (Invitrogen). DNA concentrations were adjusted to 20 ng/ul and used for GBS library preparation. GBS libraries were prepared by digestion of DNA with the *ApeK*I restriction enzyme in 96-plex, where each plate included a single random blank well. PCR amplification was performed to generate the GBS libraries, and DNA was sequenced on a Genome Analyzer II device in a single flowcell channel (Illumina Inc., USA). GBS was carried out at the University of Wisconsin-Madison Biotechnology Center as previously described (Elshire et al., 2011).

### SNP calling, filtering, and imputation

Raw reads were trimmed to remove any sequencing adapters and low-quality bases by trimming software skewer (Jiang et al., 2014). Trimmed reads were aligned to the 12x.0 version of the PN40024 reference genome (Jaillon et al., 2007; Adam-Blondon et al., 2011) using Bowtie 2 (Langmead and Salzberg, 2012), and TASSEL reference-based GBS analysis pipeline was used for SNP calling (Glaubitz et al., 2014). SNPs with minor allele frequency (MAF) > 0.05 and missing data > 0.80 at the markers and genotypes level were filtered, and monomorphic SNPs were removed. Imputation was carried out using Beagle 4.1 (Browning and Browning, 2016) with a probability > 0.80. The minor allele frequency (MAF) was calculated using TASSEL (v5.2.64) (Bradbury et al., 2007). Finally, the polymorphism information content (PIC) values were calculated using SNP data using the following equation (Botstein et al., 1980). *Pi* and Pj show the population frequency of the *i*th and *j*th allele.


$$PIC=1-\sum_{i=1}^{n}P_{i}^{2}-\sum_{i=1}^{n-1}\sum_{j=i+1}^{n}2P_{i}^{2}P_{j}^{2}$$

Observed (Ho) and expected (He) heterozygosity were calculated in the “snpReady” package (Granato et al., 2018) in R. Distributions of SNPs within 1Mb window size after filtering were visualized using the R package “CMplot” (Yin et al., 2021).

### Analysis of the genetic diversity

TASSEL (v5.2.64) (Bradbury et al., 2007) was used to calculate the kinship (centered IBS) matrix and genetic distance matrix between all 341 grapevine genotypes. Kinship heatmap and histogram were visualized using the heatmap.2 function in R package “gplots” (Warnes et al., 2009). The phylogenetic analysis was performed using the neighbor-joining method implemented in TASSEL (v5.2.64) (Bradbury et al., 2007). The phylogenetic tree was saved in Newick format in TASSEL (v5.2.64) (Bradbury et al., 2007) and visualized using the iTOL v4.3.3 online tool (Letunic and Bork, 2019).

### Analysis of population structure

A principal component analysis (PCA) was conducted using the “prcomp” function in R (Team, R.C, 2016). The PCA plots (PC1 vs. PC2, PC2 vs. PC3, PC1 vs. PC3) were constructed using the “ggplot2” package (Wickham, 2011) in R.

Population structure was investigated to infer the most likely number of ancestral populations using ADMIXTURE software version 1.3.0 (Alexander et al., 2009). Using default settings, analysis was carried out with K (number of populations) ranging from 1 to 14. The optimal number of K values was determined based on the lowest cross-validation (CV) error program reported. The admixture proportions of each genotype were visualized using the “ggplot2” package (Wickham, 2011) in R, and genotypes were assigned to a specific cluster when the estimated membership coefficient was above 0.7. Pairwise Fst (fixation index) values were calculated using ADMIXTURE software (Alexander et al., 2009). Pairwise Fst values for each SNP between the populations identified by ADMIXTURE software were also estimated using the Weir and Cockerham algorithm (Weir and Cockerham, 1984) in VCFtools 0.1.16 (Danecek et al., 2011) to identify the divergent loci. Distribution of the pairwise Fst values of each SNP across the 19 grapevine chromosomes was displayed with “ggplot2”package (Wickham, 2011) in R. SNP loci with Fst ≥ 0.5 were identified as divergent, and genes associated with divergent SNPs were found based on the PN40024 reference genome using the Jbrowse feature of Phytozome v.13 (http://phytozome.jgi.doe.gov/pz/portal.html).

Analysis of molecular variance (AMOVA) was performed to investigate the genetic differentiation within and among populations based on the defined populations by STRUCTURE and geographic origin of the genotypes using the “poppr” package in R (Kamvar et al., 2014).

### Linkage disequilibrium analysis

The level of LD was evaluated based on the squared allele frequency (*r^2^*) between SNPs on each chromosome for the entire germplasm collection using TASSEL (Bradbury et al., 2007). The SNPs were considered to be in significant LD when P < 0.01. The trend in LD decay was investigated by plotting pairwise LD values (*r^2^*) within each 300-kb bin against the distance between SNPs for each chromosome and the whole genome in R (Team, R.C, 2016). It was fitted by nonlinear regression using the expectation of *r^2^* between adjacent sites proposed by Hill and Weir (Hill and Weir, 1988).

## Results

### SNP genotyping and distribution of SNPs in the grapevine genome

**Figure 1:**
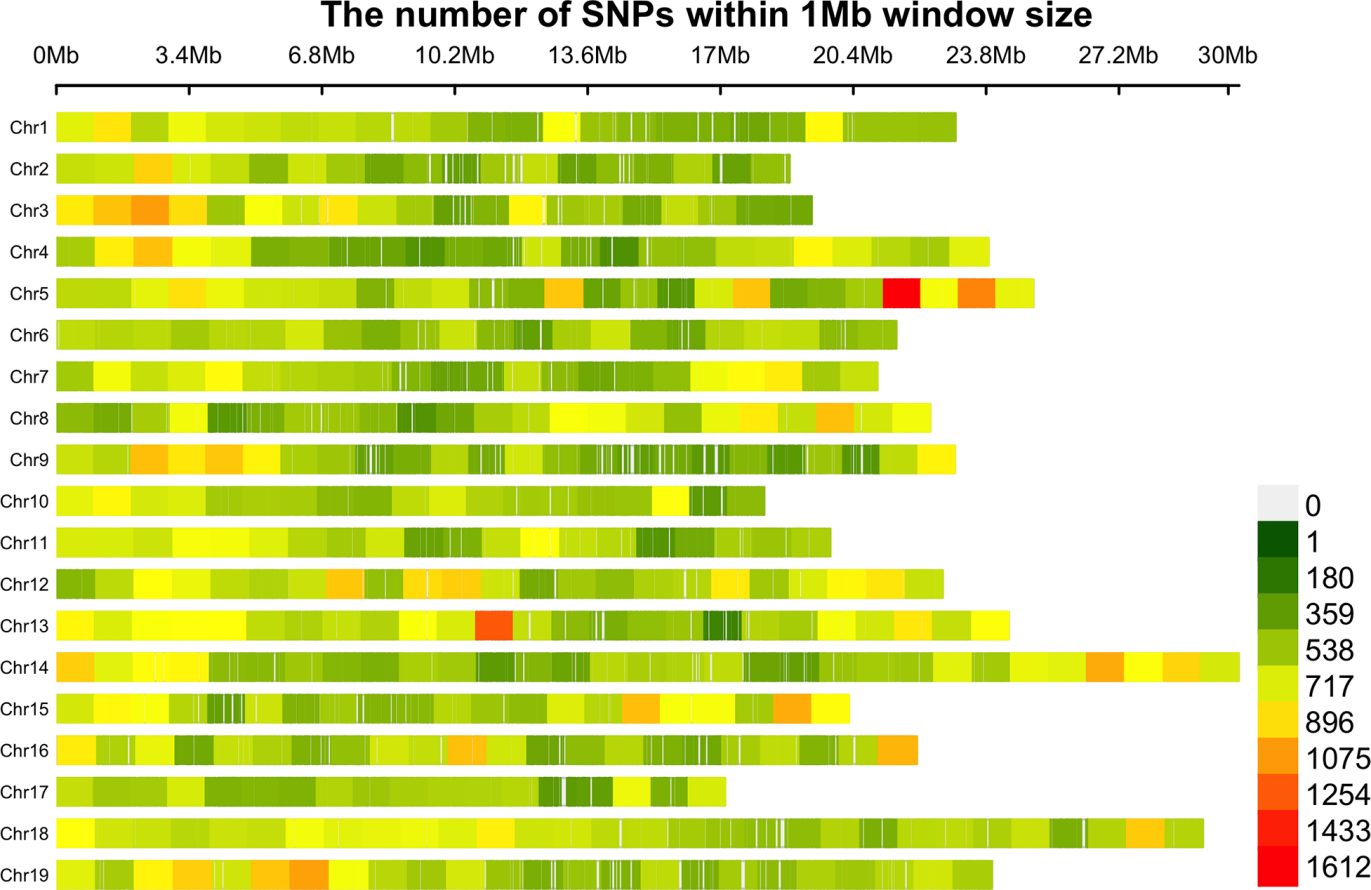
The density of SNPs on the 19 chromosomes within 1Mb window size after filtering.

**Figure 2:**
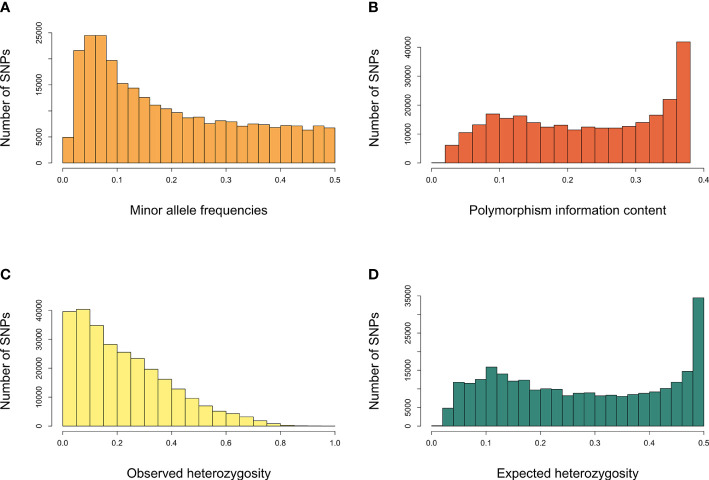
Characteristic statistics of SNPs. **(A)** minor allele frequencies, **(B)** polymorphism information content, **(C)** observed heterozygosity (Ho), **(D)** expected heterozygosity (He).

GBS sequencing of 341 grapevine genotypes generated >1 billion reads, with an average of 3,056,208 reads per sample. A set of 470,672 unfiltered SNPs was obtained from these data and 36,464 SNPs were mapped to the scaffolds not yet assigned to specific chromosomes. After filtering for minor allele frequency threshold of 0.05, >20% missing, and monomorphic SNPs, 272,962 genome‐wide SNP markers were obtained and used for downstream analysis. The number of SNPs within the 1Mb window size after filtering for each chromosome is shown in **Figure 1**. The high‐density coverage of SNPs resulted in an average of 14,366 markers per chromosome, ranging from 10,063 on chromosome 17 to 19,609 on chromosome 18. All the SNPs were mapped onto the 19 chromosomes covering a total of 425.8 Mb of the grapevine genome. As shown in **Table S2**, the chromosome size covered by SNPs ranged from 17.1 Mb (chromosome 17) to 30.3 Mb (chromosome 14). Individually, the average number of SNPs per Mb varied from 570.6 on chromosome 2 to 720.6 on chromosome 5. The average minor allele frequency was 0.196 (**Table S2**), and 85.8% of all the SNPs had MAF>5% (**Figure 2**). The polymorphic information content (PIC) values ranged from 0.0087 to 0.38, with an average of 0.23 (**Figure 2**). PIC values across 19 chromosomes were very similar, with a mean of 0.23 (**Supplementary Table S2**). The observed heterozygosity (Ho) values varied between 0.00 to 0.99, with an average of 0.23. Overall mean expected heterozygosity was higher than the observed heterozygosity. The expected heterozygosity (He) values ranged from 0.08 to 0.50, with an overall mean of 0.28 (**Figure 2**). All the raw sequencing reads for all genotypes have been submitted to the NCBI Sequence Read Archive and deposited under the “BioProject ID”: PRJNA742054.

### Genetic diversity

**Figure 3:**
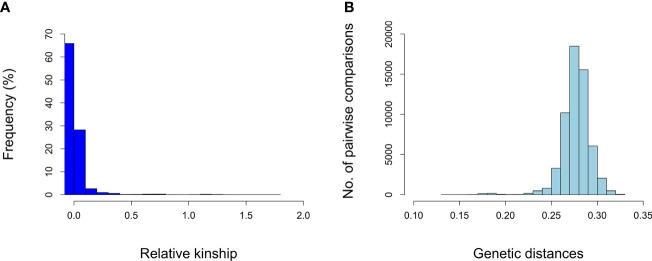
Histograms showing the distribution of pairwise relative kinship values **(A)** and distribution of pairwise genetic distances **(B)**.

A total of 272,962 SNPs on the nineteen chromosomes were used to evaluate genetic diversity in 341 genotypes. Different complementary approaches were used to estimate the genetic diversity of grapevine germplasm collection. We calculated marker-based kinship coefficients between pairs of 341 genotypes which ranged from 0.00 to 1.78 with a mean value of 0.02 (**Supplementary Table S3**). Pairwise relative kinship values of 0 accounted for 67% of all kinship coefficients. **Figure 3A** shows the distribution of the relative kinship values for all marker pairs.

The genetic diversity among grapevine genotypes was also shown by the IBS-based genetic distance matrix. The distribution of genetic distances is shown in **Figure 3B**. The genetic distance of pairwise comparisons of the grapevine genotypes varied from 0.138 to 0.325 with an average dissimilarity of 0.277 (**Supplementary Table S4**). Most of the genetic distances were between 0.25 and 0.30. The smallest genetic distance (0.138) was observed between “İnek Memesi” (c-4-3) and “Çavuş Malaga” (a-4-5). The maximum genetic distance (0.324) was found between “Kirli kadın parmağı” (a-9-3) and “Merlot” (m). For the three tested reference genotypes, the mean genetic distance was 0.301, 0279, and 0.271 for “Merlot” (m), “Cabernet Sauvignon” (cs), and “Cardinal” (c), respectively.

**Figure 4:**
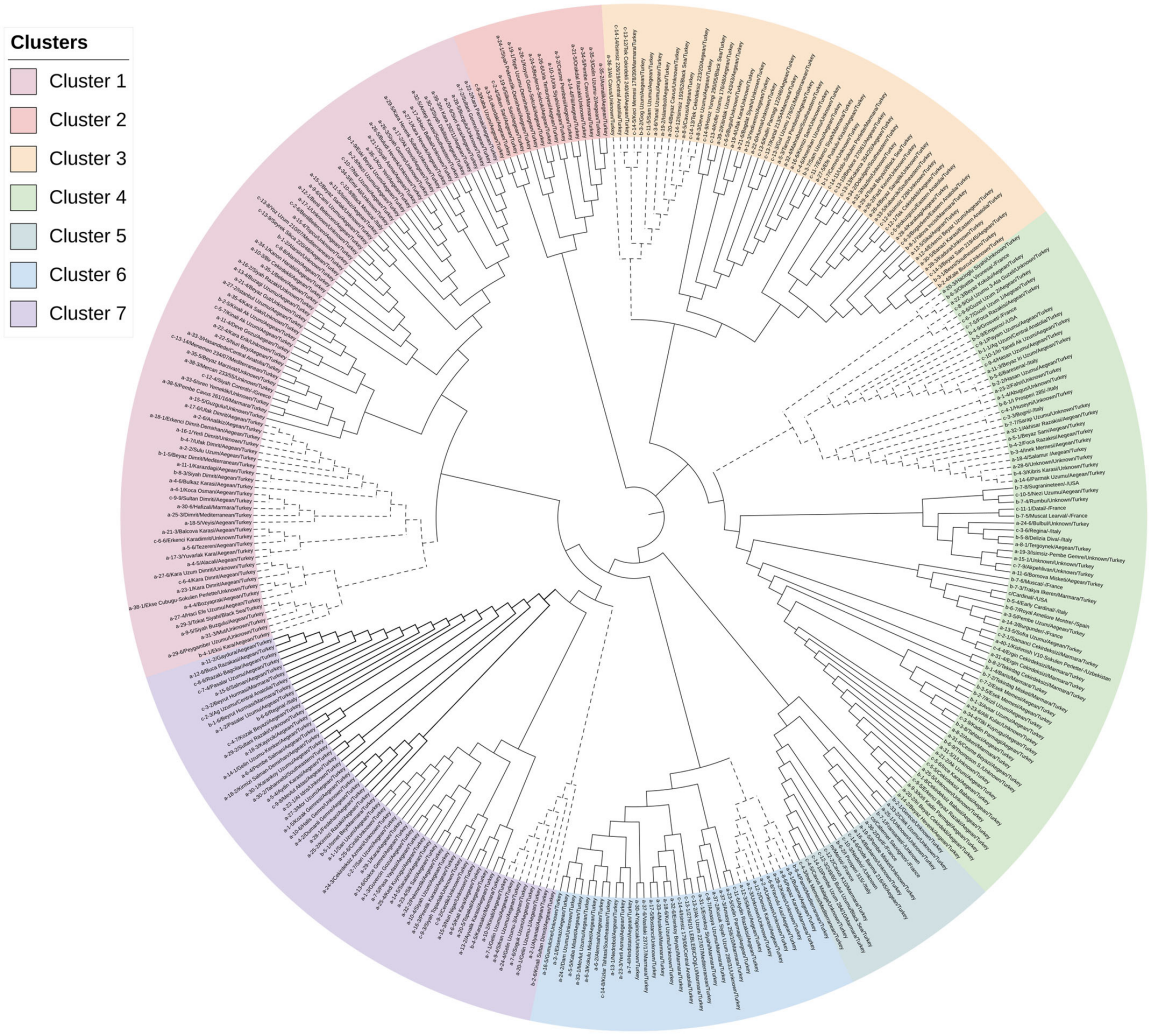
Neighbor-joining dendrogram showing the genetic relatedness among 341 grapevine genotypes.

A neighbor-joining based cluster analysis was implemented to gain further insight into the genetic diversity among grapevine genotypes. The 341 grapevine genotypes were classified into seven major clusters, each containing genotypes from different regions, indicating a high amount of genetic admixture between genotypes (**Figure 4**).

The most significant number of genotypes convened in cluster 1, containing 83 genotypes with an average genetic distance of 0.268. Cluster 1 was divided into two sub-clusters, A and B. Sub-cluster 1A included 27 genotypes from the Aegean Region, two genotypes from the Mediterranean Region, one each from the Black Sea Region, Southeastern Region, Central Anatolia Region, Italy, Greece, and 24 undefined genotypes. Sub-cluster 1B contained 31 genotypes: twenty-one from the Aegean region, two from the Mediterranean Region, one from the Marmara and the Black Sea Region, and 18 from the unknown location.

Cluster 2 (mean genetic distance=0.261) consisted of 16 genotypes, 14 from the Aegean Region, one from the Marmara region, and one from an unknown location.

Cluster 3 (mean genetic distance=0.262) contained 54 genotypes subdivided into two sub-clusters (3A and 3B). Sub-cluster 3A (n=13) had eight genotypes from the Aegean Region and one each from the Marmara Region, Central Anatolia, and the Black Sea Regions. It also contained one genotype from an unknown location. Sub-cluster 3B included 41 genotypes: fourteen from the Aegean region, four from each of the Marmara and the Southeastern Regions, three from the Eastern Anatolia Region, two from the Black Sea Region, and one from the Mediterranean Region. There were also thirteen genotypes from unknown locations in this sub-cluster.

Cluster 4 (average genetic distance=0.266) is subdivided into two sub-clusters: sub-cluster 4A and sub-cluster 4B, which consisted of 30 and 49 genotypes, respectively. Sub-cluster 4A included 30 genotypes comprising 15 genotypes from the Aegean Region, three from Italy, two from France, and one from the Central Anatolia Region and the USA. It included eight genotypes from an unknown location. Sub-cluster 4B included 49 genotypes that contained “Cardinal” (genotype c), one of the three reference grapevine genotypes. Among 49 genotypes, twenty-one were from the Aegean Region, eight from the Marmara Region, four from France, three from Italy, two from the USA, and one from Uzbekistan and Spain. Nine unknown genotypes did not have origin information.

Cluster 5 included 17 genotypes composed of 6 from an unknown location in Türkiye, two from unknown countries, three from France, two from Marmara, and one each genotype from the Aegean Region, Mediterranean Region, Black Sea Region, and Italy. These genotypes were more genetically distinct than other clusters, with an average genetic distance of 0.273. “İnek Memesi” (genotype c-4-3) and “Çavuş Malaga” (genotype a-4-5) had the lowest genetic distance (0.138) values and were placed in this cluster. In addition, “Merlot” (m) and “Cabernet Sauvignon” (cs) reference grapevine genotypes were placed in cluster 5.

Cluster 6 consisted of 35 genotypes from 5 different geographic regions of Türkiye, subdivided into two sub-clusters (6A and 6B). While sub-cluster 6A included nine genotypes from the Aegean, Marmara, and Mediterranean Regions, sub-cluster 6B consisted of 26 genotypes, including ten genotypes from the Aegean Region, seven from the Marmara, one each from the Mediterranean, Central Anatolia and Southeastern regions. Among 35 genotypes in this cluster, there were three and six from unknown locations.

There were 57 genotypes in Cluster 7 with an average genetic distance of 0.262, separated into three sub-clusters. Sub-cluster 7A included eight genotypes which were all from the Aegean Region. Sub-cluster 7B contained 21 genotypes, of which 15 were from the Aegean Region, one from the Marmara, and five from an unknown location. Sub-cluster 7C contained 28 genotypes and most of them were from the Aegean Region (n=19) and 3 genotypes from the Marmara Region, 1 each from the Central Anatolia, the Southeastern Region, and Italy and 3 genotypes from an unknown location. Although there was no clear discrimination in the phylogeny analysis based on location, this result was consistent with the model-based population structure.

**Figure 5:**
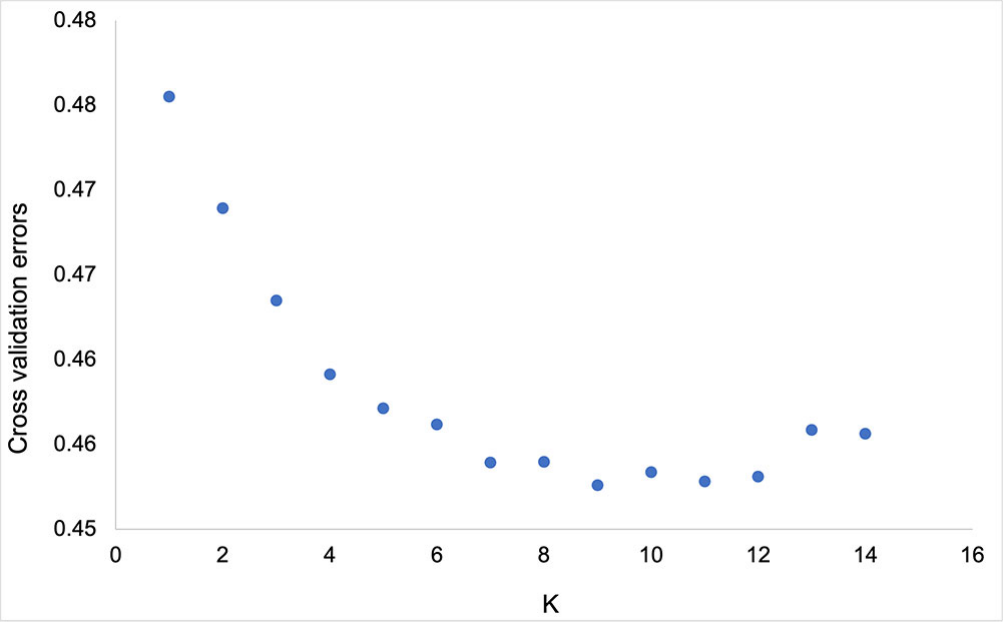
The plot of ADMIXTURE cross-validation error with different K.

**Figure 6:**
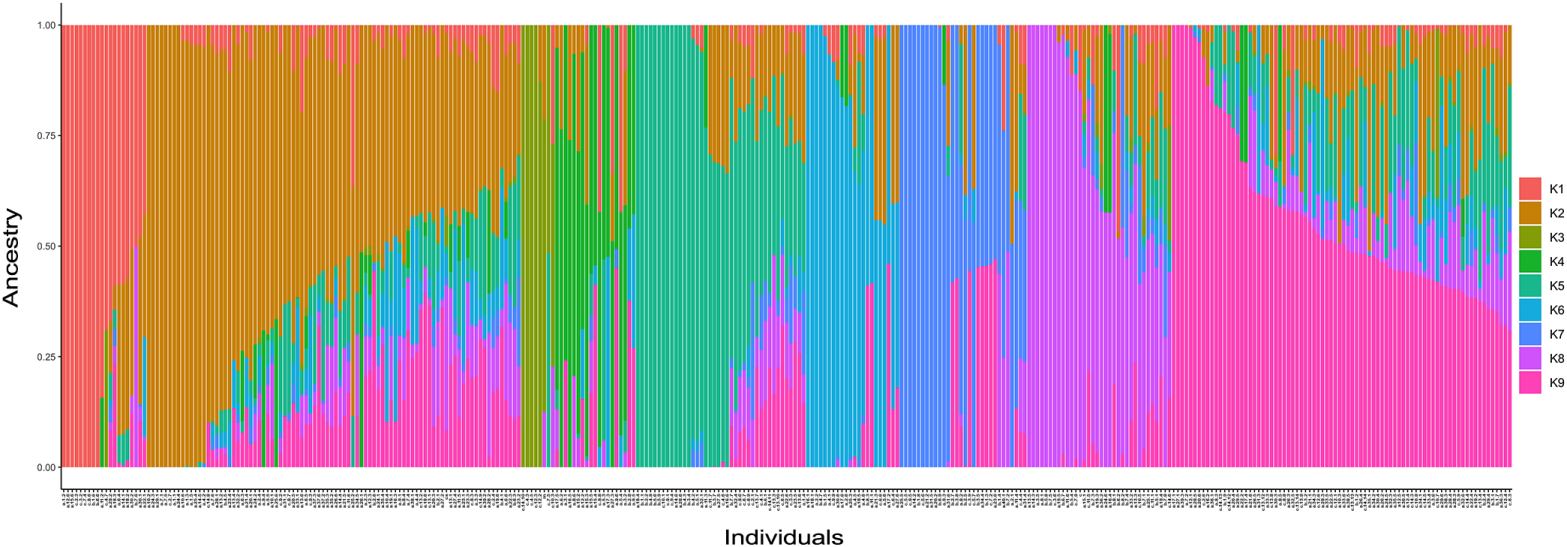
Barplot shows the group assignment by ADMIXTURE for 341 genotypes when K = 9. The plot shows individuals sorted by ‘all’ clusters.

Population structure analysis using ADMIXTURE software was performed to understand the genetic structure of 341 grapevine genotypes. The cross-validation errors were examined under the models with K = 1–14. Genotypes with an admixture coefficient higher than 0.70 were assigned to a group, while those lower than 0.7 were assigned to the admixture group. ADMIXTURE simulation showed that the minimum value of cross-validation errors was 0.45 when K=9 (**Figure 5**), indicating that grapevine genotypes were classified into nine clusters (K1-K9) based on ADMIXTURE results (**Figure 6**). The 116 assigned genotypes (approximately 34% of 341 genotypes) were structured into nine clusters, while the other 225 genotypes (66% of 341 genotypes), exhibited membership values lower than 0.70 retained in the admixed genotype cluster. Although population structure analysis did not show any evidence of clustering according to their geographic origin, some inferred clusters (K2, K3, K4, K6, K7, and K9) only contained Turkish genotypes. Most of the genotypes that originated outside of Türkiye were categorized as admixture forms with varying membership levels shared among groups. In contrast, seven, three, and one genotype from Italy, France, Spain, and the USA were located in K8, K5, and K1, respectively. Detailed information about the individual coefficient of participation in the K clusters can be found in **Supplementary Table S5**.

**Figure 7:**
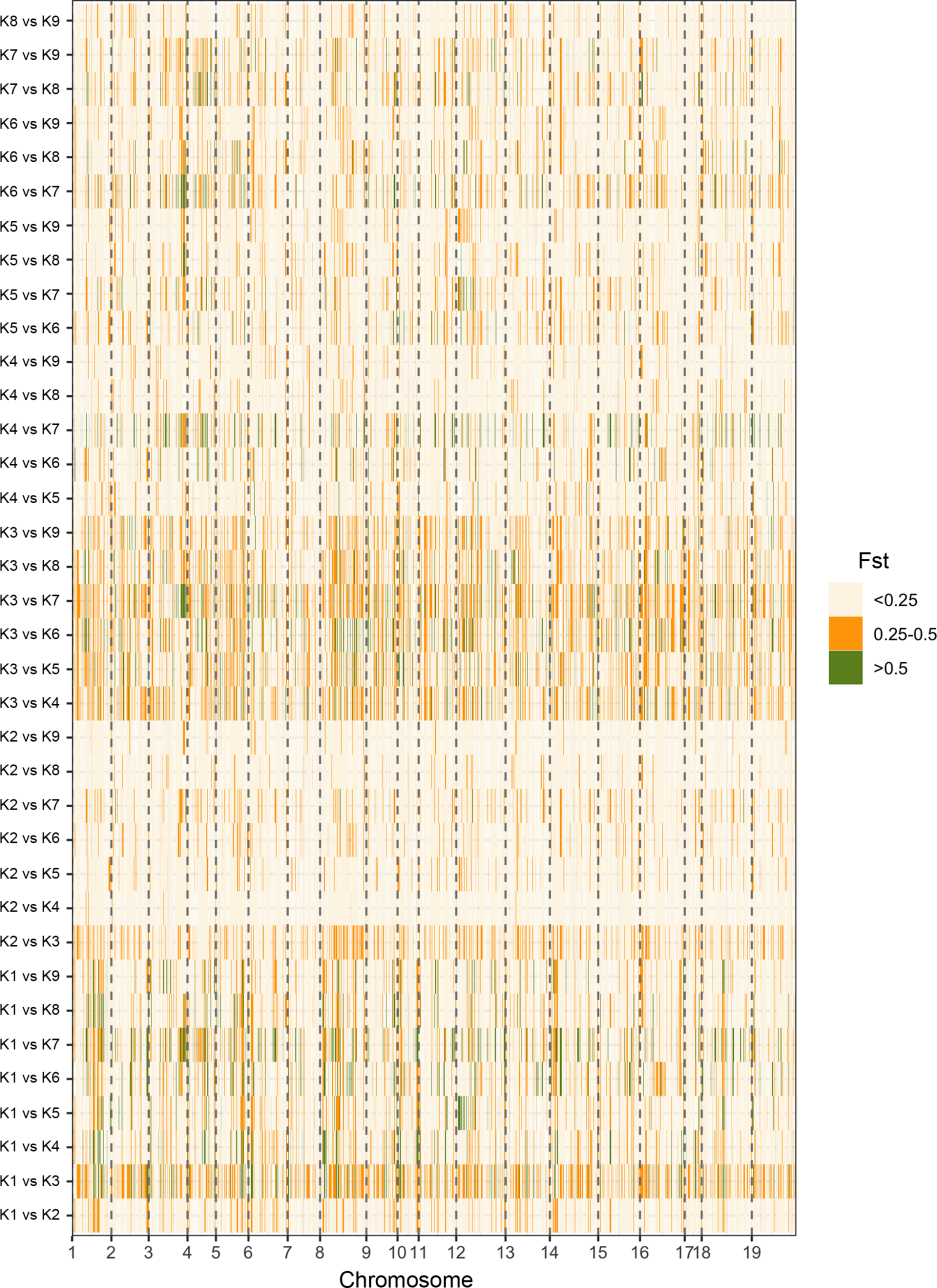
A graphical display of the Fst values for individual SNP loci between the nine populations identified by ADMIXTURE analysis.

To estimate the level of genetic differentiation, the ADMIXTURE software was used to calculate pairwise Fst values (**Table 1**). Fst values ranged from 0.077 to 0.255, with a mean of 0.15. The highest level of differentiation was observed between K1 and K3 (Fst=0.255). The lowest level of differentiation, Fst, was found between K2 and K9 (Fst=0.077). Fst values between groups showed that there was moderate divergence across all groups. In the study we also analyzed Fst values for individual SNP loci between the nine populations identified by ADMIXTURE analysis (**Figure 7**). Using a threshold of Fst >0.5, a total of 3091 divergent loci were detected (**Table S6**). The K2 vs K7 comparison had the lowest number (14) of divergent loci, while the highest (350) was observed in K1 vs K7. Among these, 1889 SNPs (61%) were found associated with genes. Forty-six highly divergent loci (FST values = ~ 0.93) identified between K3 and K7 and thirty-four of them were associated with a gene which is related important metabolic processes such as LRR and NB-ARC domains-containing disease resistance protein, heavy metal transport/detoxification superfamily protein, GATA type zinc finger transcription factor family protein, and AGD2-like defense response protein 1 (**Table S6**).

**Table 1:** Fst values between estimated populations.

Cluster	K1	K2	K3	K4	K5	K6	K7	K8	K9
K1	0								
K2	0.122	0							
K3	0.255	0.195	0						
K4	0.173	0.103	0.24	0					
K5	0.148	0.091	0.212	0.136	0				
K6	0.171	0.11	0.236	0.145	0.127	0			
K7	0.193	0.135	0.251	0.181	0.135	0.17	0		
K8	0.144	0.079	0.205	0.13	0.097	0.125	0.145	0	
K9	0.137	0.077	0.202	0.115	0.088	0.104	0.132	0.091	0

To understand the sources of genetic differentiation within and among the populations in the germplasm collection, we conducted an AMOVA based on the defined populations by structure analysis and geographic origin of the genotypes. The structure-based AMOVA results indicated that most of the observed genetic variation could be attributed to differences within populations (99.37%) rather than to the variation among populations (0.63%). Similarly, origin-based AMOVA also showed that the between population component of genetic variance is 0.65% and within population is 99.35% (**Table 2**).

Table 2Analysis of molecular variance (AMOVA) for 341 grapevine genotypes by grouping them based on STRUCTURE software and their geographic origins.

**Table:** 

Structure-based AMOVA					
	df	Sum Sq	Mean Sq	Var	%
Among populations	9	909560.2	101062.24	584.84	0.65
Within populations	331	29428197.3	88906.94	88906.94	99.35
Total	340	30337757.4	89228.7	89491.78	100

**Table:** 

Origin-based AMOVA					
	df	Sum Sq	Mean Sq	Var	%
Among populations	13	1282965	98689.60	584.84	0.63
Within populations	327	29054793	88852.58	88906.94	99.37
Total	340	30337757	89228.70	89491.78	100

df, degrees of freedom; Sum Sq, Sum of squares; Mean Sq, Mean square; Var, Estimated variance.

**Figure 8:**
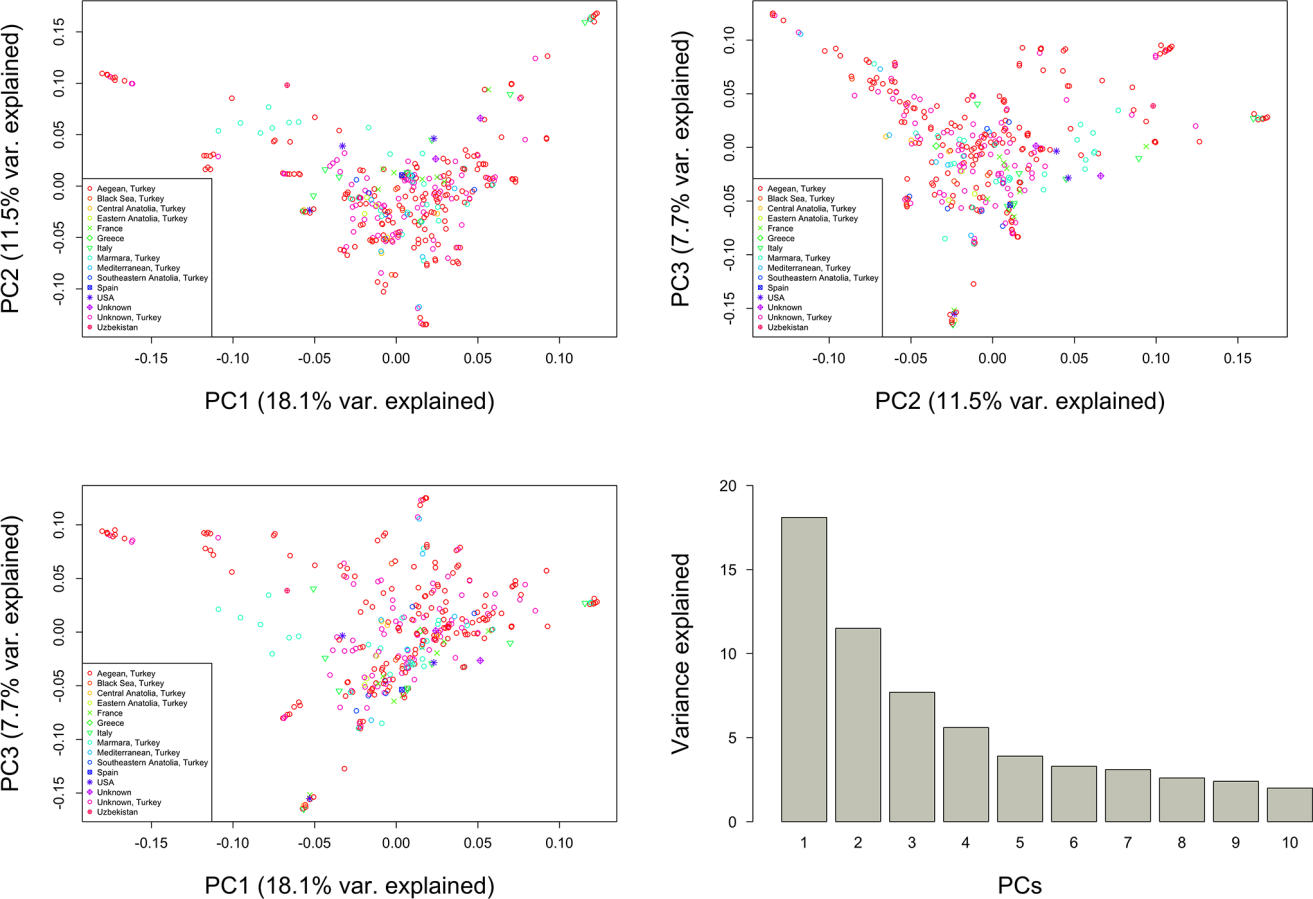
Principal Components (PC) 1, 2, and 3 based on 272,962 genome‐wide SNPs scored on 341 grapevine genotypes.

The first three principal components (PCs) accounted for 37% of the genetic variation observed in the data (**Figure 8**). The first PC accounted for 18.1% of the observed variation, while the second and third PCs contributed 11.5% and 7.7%, respectively. Most genotypes from different origins showed no clear separation across germplasm collection (**Figure 8**).

### Linkage disequilibrium

**Figure 9:**
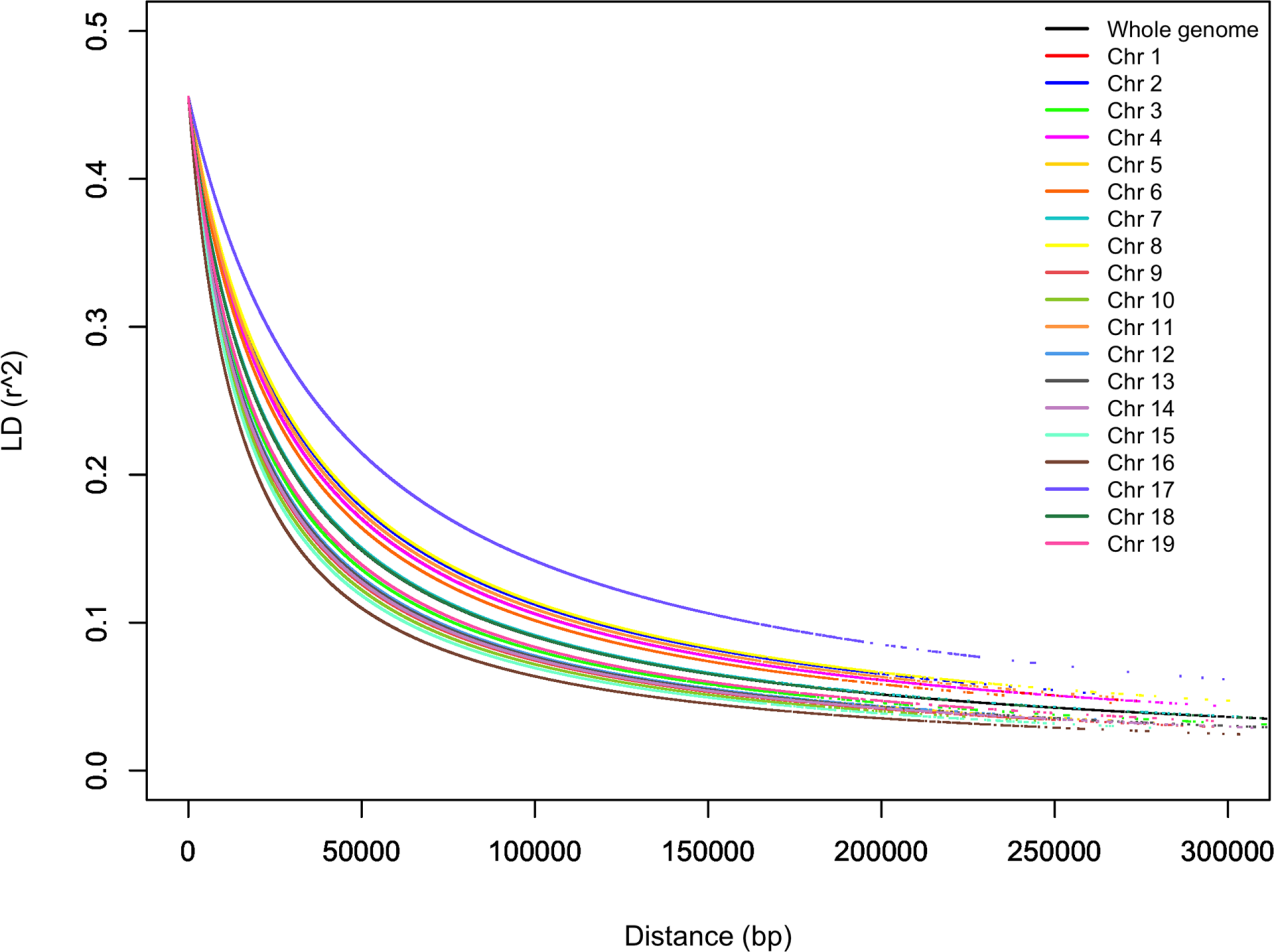
Linkage disequilibrium (LD)-measured *r^2^* plotted vs. the physical map (bp) between pairs of SNP markers.

LD was estimated for each pairwise SNP combination in the entire germplasm collection. A total of 2.481,581 (20.1%) pairs of markers showed a significant LD value (*r^2^*) at p < 0.01, while 1.901,084 (15.4%) pairs of markers showed a significant LD at P < 0.001. The *r^2^* values for all significant loci ranged from 0.019 to 1, averaging 0.29. Only 131,151 (1.06%) pairs of markers showed complete LD (*r^2 = ^* 1). LD decay varied across the 19 chromosomes, as shown in **Figure 9**. LD in chromosome 16 decayed the most rapidly and slowly in chromosome 17. In general, LD decayed very fast when *r^2^* was between 0.45 and 0.2 and became flat when *r^2^* was 0.05 (**Figure 9**). The average LD decay for the entire genome was 30 kb when *r^2 = ^* 0.2. LD decay for a predicted *r^2^* of 0.2 varied from 20 to 60 kb based on the chromosomes.

Average pairwise *r^2^* values were similar among different chromosomes, ranging from 0.273 (chromosome 10) to 0.310 (chromosome 17) (**Supplementary Table S7**). Therefore, a number of significant and non-significant LD pairs and average *r^2^* values for them were given for each chromosome in **Supplementary Table S7**.

## Discussion

The key requirements for progress in sustainable grapevine breeding programs depend upon the exploitation of genetic resources and characterization of genetic diversity as well as the investigation of genotype-phenotype associations by genomic and molecular techniques. For this purpose, GBS technique provides a large number of SNP markers distributed throughout the genome to enhance our knowledge of the genetic structure and genetic variability within grapevine germplasm collections.

The main objective of this study was to explore the genetic diversity and population structure of 341 grapevine genotypes from grapevine germplasm collection at Manisa Viticulture Research Institute using GBS-generated SNP markers. To our knowledge, no studies have been performed yet on Turkish grapevine germplasm based on high-throughput SNP discovery. This study is also the first comprehensive report in which the genetic diversity, population structure, and characterization of Turkish genotypes.

### Genotyping by sequencing

High-throughput and cost-effective SNP discovery approaches such as GBS have become the most popular genotyping technique in plant species. They can be effectively used for various purposes in plant breeding and genetics (Elshire et al., 2011). In this study, GBS protocol enabled the discovery of thousands of SNPs to discriminate the 341 grapevine genotypes by providing many advantages, including relatively low cost, quick sample handling, and reduced genome complexity (Elshire et al., 2011; Barba et al., 2014). So far, several studies have used the GBS in domesticated and wild grapevine to assess genetic diversity (Klein et al., 2018; Miazzi et al., 2019; Miazzi et al., 2020), clarify evolutionary relationships (Klein et al., 2018), and identify marker-trait associations by QTL (Barba et al., 2014) and genome-wide association mapping (Guo et al., 2019).

Our study used one-enzyme (*ApeK*I) based GBS to genotype. *ApeK*I has comparatively few recognition sites in the major classes of plant retrotransposons, which helps reduce genome complexity (Elshire et al., 2011). Similarly, various studies preferred *ApeK*I enzyme to perform GBS in grapevine (Barba et al., 2014; Klein et al., 2018; Guo et al., 2019; Tello et al., 2019b). The average number of 3.1 million sequence reads per sample obtained in this study was much higher than other GBS studies in grapevine (Barba et al., 2014; Klein et al., 2018; Tello et al., 2019b). Klein et al. (Klein et al., 2018) reported 1.7 million sequence reads per sample by GBS of 359 Vitis genotypes. Averages of 712 thousand sequence reads per sample, and 2.8 million sequence reads per sample were obtained from the Illumina GA3 and HiSeq systems, respectively, by Barba et al. (Barba et al., 2014). Guo et al. (Guo et al., 2019) reported the highest number of sequence reads per sample, 4.8 million, in grapevine genotypes. These differences obtained from various studies were mainly due to different grapevine collections and the sequencing platform used for GBS. The average number of sequence reads per sample that was obtained here was also similar to what has been reported for various tree species: 3.5 million for apricot (Gürcan et al., 2016), 2.6 million for olive (Kaya et al., 2019), and 2.4 million for peach (Bielenberg et al., 2015).

After filtering based on missingness and MAF, a total of 272K high-quality SNPs were obtained, which is higher than the number of SNPs detected in other GBS studies in the grapevine (Barba et al., 2014; Klein et al., 2018; Guo et al., 2019; Miazzi et al., 2020). Previously published studies in grapevine have produced a varying number of SNPs after filtering, such as 32K SNPs in 179 cultivars (Guo et al., 2019), 17K SNPs in 85 hybrid genotypes (Barba et al., 2014), 11K SNPs in 359 genotypes (Klein et al., 2018), and 25K SNPs in 40 genotypes (Miazzi et al., 2020) by GBS. In this study, high-quality SNPs covered all 19 chromosomes, and the number of SNPs per chromosome correlated highly with the chromosome size. A similar pattern of SNP distribution was reported by Guo et al. (Guo et al., 2019).

PIC is affected by marker types in addition to genetic diversity, genotype size, and breeding type of the species (Singh et al., 2013). While the PIC value of SNPs ranges from 0 to 0.5 due to the bi-allelic nature of the marker, it can be as high as 0.5–1.0 for multiallelic SSR markers (Singh et al., 2013; Chen et al., 2017). In this study, PIC values ranged from 0.0087 to 0.38, with an average value of 0.23. PIC value in a similar range was reported for SNP markers in a collection of grapevine genotypes by Cabezas et al. (Cabezas et al., 2011), Mihaljevic et al. (Žulj Mihaljević et al., 2020), and Lijavetzky et al. (Lijavetzky et al., 2007). Higher PIC values were reported in other grapevine studies with SSR markers (De Oliveira et al., 2020; Miazzi et al., 2020). Although the low PIC value of SNP markers is a limitation, it can be overcome by using a large number of SNPs (Lijavetzky et al., 2007). Although PIC values of SNP markers indicated low to moderate discriminating power in the study, 272K high-quality SNPs successfully assessed genetic diversity and investigated of genetic relatedness of the grapevine genotypes.

Observed heterozygosity (Ho) of the SNPs across 341 grapevine genotypes varied from 0.00 to 0.99 with an average of 0.23, which was slightly lower than the expected heterozygosity (Ho) that ranged from 0.08 to 0.50, with an average of 0.28, confirming the high level of heterozygosity in grapevine. The Ho range observed in this study was more comprehensive in range than 0.48 to 0.88 reported by Oliveira et al. (De Oliveira et al., 2020), 0.364 to 0.755 by Zdunic et al. (Zdunić et al., 2020), 0.32 to 0.94 by Mihaljevic et al. (Žulj Mihaljević et al., 2020). Mihaljevic et al. (Žulj Mihaljević et al., 2020) investigated the genetic diversity of the grapevine genotypes grown in Croatia and showed a mean observed heterozygosity of 0.71, greater than the mean expected 0.23 in this study. Furthermore, higher overall mean Ho values were obtained among cultivated grapevines in Portuguese (Ho= 0.361) (Cunha et al., 2020), Tunisia (Ho=0.325) (Ghaffari et al., 2014), Italy (Ho=0.791) (D’Onofrio, 2020), and various European countries from Central Europe and Part of the Western Balkan Peninsula (Ho= 0.578) (Zdunić et al., 2020). The He value obtained in this study was comparable with 0.304 and 0.348 reported by Ghaffari et al. (Ghaffari et al., 2014) and Cunha et al. (Cunha et al., 2020), respectively, while it was lesser than 0.698, 0.7, 0.777 reported by Zdunic et al. (Zdunić et al., 2020), Mihaljevic et al. (Žulj Mihaljević et al., 2020) and D’Onofrio (D’Onofrio, 2020). In line with our study, the expected heterozygosity value obtained from biallelic SNP markers (Ghaffari et al., 2014; De Lorenzis et al., 2019; Cunha et al., 2020) was lower than that of the studies which used multiallelic markers such as SSRs (Augusto et al., 2021; Péros et al., 2021; Cretazzo et al., 2022).

### The assessment of genetic diversity and population structure of the grapevine genotypes

For efficient conservation and utilization of germplasm collections, genetic diversity and population structure characterization have great significance (Ramanatha Rao and Hodgkin, 2002). Previous studies have been conducted on grapevine genetic characterization in the most important grapevine germplasm collections (Maul and Töpfer, 2015; Žulj Mihaljević et al., 2020). In this study, we have attempted to characterize 341 genotypes from grapevine germplasm collection in Manisa Viticulture Research Institute using GBS technology to enhance the conservation, development, and utilization of genetic resources in Türkiye.

In recent years, Viticulture Research Institutes in Türkiye significantly contributed to the conservation and use of Turkish grapevine germplasm collections. Ampelographic descriptors have characterized some important genotypes (Ates et al., 2011; Atak et al., 2014; Kara et al., 2018). Despite its high importance and implemented conservation strategies in Viticulture Research Institutes, molecular characterization of Turkish genotypes lagged behind other grape-producing countries. In the past, various grapevines, including both cultivated and wild genotypes from different geographical regions of Türkiye, have been analyzed using SSR markers to estimate the genetic diversity and structure (Vouillamoz et al., 2006; Selli et al., 2007; Tangolar et al., 2009; Boz et al., 2011; Yılmaz et al., 2020). A research project aimed to characterize 1150 Turkish genotypes in Tekirdağ Viticulture Research Institute by SSR markers; however, its results are not published (Gökbayrak and Söylemezoglu, 2010).

This is the first study in which the genetic diversity, structure, and characterization of 341 genotypes from Turkish germplasm collection, including national and international varieties such as Merlot, Cabernet Sauvignon, and Cardinal, were compared using GBS markers. Analyzed genotypes have agronomic and economic importance for the viticulture industry worldwide.

This study used complementary approaches to explore genetic diversity and population structure among grapevine genotypes. IBS-based genetic distance matrix and marker-based kinship coefficients were obtained to analyze genetic diversity using the three complementary clustering methods: Neighbor Joining (NJ)-based hierarchical clustering, Principal Coordinate Analysis (PCA), and Bayesian model-based clustering. In our study, the genetic distance coefficient ranged from 0.138 to 0.325, with an average dissimilarity of 0.277. The results showed that the 341 grapevine genotypes possessed a high genetic variation. Kinship values also demonstrate high genetic variation among most grapevine genotypes studied. The genetic dissimilarity values obtained in this study are comparable to those in other studies to characterize of Turkish grapevine genotypes. Yılmaz et al. (Yılmaz et al., 2020) investigated 88 grapevine genotypes from the Central Anatolia region in Türkiye using SSR markers and reported genetic distances ranging from 0.056 to 0.207. Different genetic similarity/dissimilarity values among Turkish genotypes have been reported from different groups (Tangolar et al., 2009; Boz et al., 2011), which can be explained by the smaller size of the genotypes and different types of markers used in comparison with our study.

In this study, the neighbor-joining-based cluster analysis was implemented without considering the origin of the grapevine genotypes. Although grapevine genotypes were classified into seven major clusters and some genotypes were clustered as sub-clusters, the clustering pattern did not reflect the geographical origin of samples providing the common genetic background. Similar to our results, cluster analysis carried out in several other studies could not classify grapevine genotypes based on geographic origin (Ghaffari et al., 2014; De Lorenzis et al., 2019; Miazzi et al., 2020). However, Lorenzis et al. (De Lorenzis et al., 2015) and Zdunic et al. (Zdunić et al., 2020) were able to differentiate wild and cultivated grapevine genotypes with cluster analysis. Furthermore, differentiation between Western and Eastern European cultivars in phylogenetic trees was also shown by Zdunic et al. (Zdunić et al., 2020).

The PCA and structure analysis did not distinguish between the grapevine genotypes of different origins, highlighting the occurrence of gene flow and a high amount of admixture. Our results agree with Cipriani et al. (2010) for a collection of 1005 grapevine genotypes, reporting a weak correlation with their geographical origin based on the PCA. Several studies reported that PCA could separate wild and cultivated genotypes from different regions. Zdunić et al. (2020) conducted PCA based on 20 SSRs among 243 genotypes sampled in several European countries and obtained two major groups corresponding to the wild and cultivated grapevines. In another study, while PCA could distinguish 57 Tunisian grapevines into wild and cultivated genotypes, the groups did not seem to represent the geographic origins of the genotypes (Ghaffari et al., 2014).

In this study, 3091 divergent loci (Fst value>0.5), including 49 highly divergent SNP loci (Fst value>0.9), were identified by the estimation of pairwise FST values. The genes with the highest Fst value are involved in a wide variety of cellular processes, including transportation and detoxification of heavy metal ions (VIT_201s0011g04710), defence response of plants against infection (VIT_212s0055g00920) and biosynthesis of secondary metabolites and the regulation of gene expression (VIT_206s0004g07350) (**Table S6**). We identified the “VviERF045” gene (VIT_203s0063g00560, Fst value=0.93), which belongs to the Ethylene Responsive Factor (ERF) family of transcription factors. VviERF045 is involved in the regulation of fruit ripening and response to biotic and abiotic stresses in grapevine {Leida, 2016, Insights into the role of the berry-specific ethylene responsive factor VviERF045}. Divergent loci associated with VIT_203s0063g00560, which encodes a protein, were annotated as a cadmium/zinc-transporting ATPase in regulating cadmium uptake and transport in grapevine. Previously, Miazzi et al.,{Miazzi et al., 2020, Marginal grapevine germplasm from Apulia (Southern Italy) represents an unexplored source of genetic diversity} identified divergent loci associated with genes involved in nitrogen metabolism, plant development, defense response, ripening process, stomal movement, and carbohydrate metabolic processes. Similarly, Marrano et al. {Marrano et al., 2018, Genomic signatures of different adaptations to environmental stimuli between wild and cultivated Vitis vinifera L} also identified divergent loci associated with genes involved in the same metabolic pathway reported by Miazzi et al. {Miazzi et al., 2020, Marginal grapevine germplasm from Apulia (Southern Italy) represents an unexplored source of genetic diversity} In addition, several divergent loci, which were significantly differentiated between sativa and sylvestris populations found to be associated with genes involved in the adaptation to environmental changes {Marrano et al., 2018, Genomic signatures of different adaptations to environmental stimuli between wild and cultivated Vitis vinifera L}.

Our study found that AMOVA results indicated a high level of genetic differentiation within populations, while variation among populations was extremely low. This suggests that the high degree of differentiation within the population may be due to high levels of genetic exchange or gene flow. These findings are consistent with previous studies on grapevines that found high levels of diversity within populations but low levels of genetic diversity among populations (Ergül et al., 2011; Imazio et al., 2013; Riaz et al., 2018).

Since somatic mutations have more chance to happen and accumulate throughout the lifespan of perennial fruit crops, they create an important source of genetic variation (Torregrosa et al., 2011). Vegetative propagation is used to stabilize and propagate the grapevine somatic variants and vegetative descendant of a grapevine are called “clones” (Ibáñez et al., 2015). Although most clones of the same variety are identical, some can show phenotypic and genotypic differences over time (Roach et al., 2018). Phenotypic differences in the clones of the same cultivar have been reported in many studies (Roach et al., 2018; Grimplet et al., 2019; Vondras et al., 2019). Although clones are considered part of the same variety based on phenotypic characteristics, there is no clear distinction between clones and cultivars (Pelsy, 2010). Cases of synonymy (one genotype with several denominations) and homonymy (one denomination for several genotypes) can result from the clones of the same variety with phenotypic differences due to the somatic mutations and the mislabeling during the propagation of grapevines, respectively (Walker et al., 2006; De Oliveira et al., 2020). As in other grapevine-growing countries, the presence of homonyms and synonyms among local varieties has been reported in Turkish genotypes by ampelography and amperometry analysis (İşçi and Altındişli7 2017; Arif et al., 2012) and molecular markers (Boz et al., 2011; Yılmaz et al., 2020). In our study, there are some genotypes with the same name that are adjacent to each other such as the two “Alarşın” (b-1-2 and c-8-8), the two “Ergin Çekirdeksizi” (a-31-4 and c-4-4), the two “Eşek Memesi” (c-7-2 and b-3-5), the two “Kara Dimrit” (a-23-1 and c-6-4), the two “Kınalı Ak Üzüm” (c-5-7 and b-2-5), the two “Silken Sarı” (a-19-6 and c-2-4). Although these genotypes were adjacent to each other, they had an apparent genetic distance value suggesting that they are homonymous. A few genotypes are also very close to each other in the same cluster which has the same name, including the two “Benli Belercen” (c-2-6 and a-12-1), the two “Beyrut Hurması” (b-1-6 and c-3-2), the two “Çekirdeksiz Babası” (c-5-5 and b-1-8), the two “Hasan Üzümü” (c-9-4 and b-2-2), the two “Ufak Dimrit” (a-17-6 and b-4-7). Although the two “Paşalar Üzümü” (a-1-2 and c-7-4) are in the same cluster, they are not close to each other. Many genotypes, such as the two “Foça Razakısı” (c-7-5 and b-4-2), the two “İnek Memesi” (b-3-4 and c-4-3), the two “Nezi Üzümü” (b-2-8 and c-10-5), the two “Şam Üzümü” (b-3-7 and c-11-5) and the two Regina (b-6-6 and c-3-6) have same names but different origins and did not cluster in the dendrogram and seemed to be homonymous. Possible synonyms and homonyms in Turkish grapevine germplasm have been previously reported by Ergül et al. (Ergül et al., 2006), Vouillamoz et al. (Vouillamoz et al., 2006), Tangolar et al. (Tangolar et al., 2009), Boz et al. (Boz et al., 2011), İşçi et al. (Burçak and DİLLİ, 2015) and Yılmaz et al. (Yılmaz et al., 2020). Genotyping in these studies is mainly based on SSR markers which are very useful for genetic diversity evaluation and cultivar identification, including the detection of synonyms and homonyms (Cipriani et al., 2010; Laucou et al., 2011; Emanuelli et al., 2013). The genetic diversity among the central Anatolian grapevine genotypes was recently characterized using SSRs by Yılmaz et al. (Yılmaz et al., 2020). Their study reported two cases of identical, seven cases of homonymous, and nine cases of synonymous grape genotypes based on 17 SSR markers (Yılmaz et al., 2020). In another study, one synonym and four homonymies were identified among 55 grape cultivars originating from the Southeastern Region using 14 SSR markers (Boz et al., 2011). Although SSR markers are highly informative, reproducible, codominant, and multi-allelic, their limitations in the accurate identification of clones have been also reported (González-Techera et al., 2004; Pelsy et al., 2010; Mercati et al., 2016). Homonym and synonym cultivars were also reported in Spanish (Moreno-Sanz et al., 2008), Iranian (Fatahi et al., 2003), Armenian (Margaryan et al., 2021), and Italian (Cipriani et al., 2010) cultivars.

### Linkage disequilibrium

LD is the non-random association between alleles at different sites and depends on many factors such as recombination, mutation, genetic drift, selection, and population admixture and size (Flint-Garcia et al., 2003). While LD decays more rapidly in cross-pollinated plants, self-pollinated plants show less decay of LD related to less effective recombination (Campoy et al., 2016). In this study, a comprehensive genome-wide LD in grapevine was evaluated by high-density SNPs (272K) over 341 genotypes. *r^2^* values decreased to 0.2 within 30 kb and 0.1 within 90 kb for the whole genome. This result is expected and consistent with the trend of rapid LD decay in grapevines (Myles et al., 2011; Nicolas et al., 2016; Guo et al., 2019). The First LD study in grapevine was reported by Barnaud et al. (Barnaud et al., 2006) using SSR markers. Up to 16.8 cM large extent of LD was observed within linkage groups. Myles et al. (Myles et al., 2011) observed that the decay of LD was down to 0.2 within less than 10 kb using the Vitis9K SNP array. Lijavetzky et al. (Lijavetzky et al., 2007) evaluated the LD decay with the 1500 SNPs and observed a lower level of LD which *r^2^* values decreased to 0.2 within 250 bp. In another study, LD reached 0.2 within 10kb using 26K SNPs (Marrano et al., 2018). Guo et al. (Guo et al., 2019) observed faster LD decay which *r^2^* reached 0.03 within 16.6 kb using 32K SNPs by GBS. In our study, significant variation in LD decay was found among chromosomes; the decay of LD was down to 0.2 ranged from 20 to 60 kb. Nicolas et al. (Nicolas et al., 2016) obtained variability in LD decay changing based on the subgroups and genomic regions. LD decay reached 0.2 and varied from 9 to 458 kb (Nicolas et al., 2016).

To assess LD in wild *V. vinifera*, 85 plants from Southern France were analyzed by 36 SSR markers, and LD was slower in cultivated than in wild grapevine (Barnaud et al., 2010). It was reported that this difference between cultivated and wild grapevine is derived from the vegetative propagation and domestication bottlenecks (Barnaud et al., 2010). By contrast, Myles et al. (Myles et al., 2011) reported a consistent trend of rapid LD decay between wild and domesticated grapevine. Marrano et al. (Marrano et al., 2017) also analyzed wild and domesticated grapevines using 14K high-quality SNPs by RAD sequencing. Vitis20K SNP array was also used for genotyping of the same grapevines by Marrano et al. (Marrano et al., 2018), and they combined SNPs (26K) from both RAD sequencing and the Vitis20K array for LD analysis. In contrast to Barnaud et al. (Barnaud et al., 2010) and Myles et al. (Myles et al., 2011), slower LD decay was observed within wild grapevines, which *r^2^* reached values below 0.2 within 20kb, LD (*r^2^*) decayed below 0.2 within 10 kb in domesticated grapevines based on RAD sequencing. When they used combined data (26K), the decay of LD appeared slower within the wild group, where *r^2^* reached values below 0.2 within 20 kb (Marrano et al., 2018).

These discrepancies could be attributed to different types or varying numbers of markers that affect the genome’s coverage (Marrano et al., 2018). In addition, since different factors such as mutation, recombination, selection, mating system, and population contribute to LD, variation in LD estimates may also be related to these factors (Flint-Garcia et al., 2003).

## Conclusion

This study used high-throughput GBS technology to explore genetic diversity, population structure among grapevine genotypes, and the possibility of using SNP markers for genetic analyses in genetic enhancement. The hierarchical cluster and principal components analysis indicated a mixing of grapevines from different regions, suggesting the strong gene flow among genotypes. This is in line with the results of population structure analysis which showed a high amount of genetic admixture in Turkish genotypes. Information on genetic diversity and population structure is essential to provide valuable genetic resources for grapevine genetic improvement and protect and develop this vital crop from erosion. Furthermore, extensive genetic diversity should be identified and maintained for a sustainable viticulture industry to develop new grape cultivars with desired traits through marker-assisted breeding. Identifying divergent loci could provide useful information for developing grapevine varieties that adapt effectively to new environmental conditions.

## Data availability statement

All the raw sequencing reads for all genotypes have been submitted to the NCBI Sequence Read Archive and deposited under the “BioProject ID”: PRJNA742054.

## Author contributions

HK, YD, and AÜ conceived and designed the study. HK, YD, and TO performed the experiments. HK analyzed the data and prepared figures and tables. All authors contributed to the article and approved the submitted version.
